# Atypical Neuroleptic Malignant Syndrome Associated with Use of Clozapine

**DOI:** 10.1155/2017/2174379

**Published:** 2017-02-20

**Authors:** Quevedo-Florez Leonardo, Granada-Romero Juliana, Camargo-Arenas Juan Fernando

**Affiliations:** Emergency Department, Hospital Universitario San Ignacio, Pontificia Universidad Javeriana, Bogotá, Colombia

## Abstract

The Neuroleptic Malignant Syndrome (NMS) is a medical emergency of infrequent presentation in the emergency department, which is associated with the use of psychiatric drugs, such as typical and atypical antipsychotics. Our case addresses a 55-year-old patient diagnosed with undifferentiated schizophrenia for 10 years, who had been receiving clozapine and clonazepam as part of their treatment. This patient presents the symptoms of Neuroleptic Malignant Syndrome without fever, which improves with treatment especially with the withdrawal of clozapine. In the absence of fever and clinical improvement, the patient is considered to have an atypical presentation of this disease.

## 1. Introduction

The Neuroleptic Malignant Syndrome (NMS) is a medical emergency of rare presentation in a service. Its appearance is associated with the use of a group of drugs frequently used in psychiatry, within which the less frequent ones in developing this disease are atypical antipsychotics such as clozapine. This disease is a fatal syndrome that, despite its clinical symptoms, which is easily recognizable by having a classic presentation, occasionally does not present all the described characteristics; this ignorance and low clinical suspicion lead to delayed diagnosis that can result in progression and fatal outcomes like death.

## 2. Case

This is a 55-year-old male patient institutionalized in a psychiatric clinic 10 years ago, diagnosed with undifferentiated schizophrenia in management with clozapine 500 mg day and clonazepam 6 drops a day, admitted to the emergency room due to altered state of consciousness, disorientation, and decreased production of language, associated with rigidity, with no other symptoms, for one week (37.2°C). Physical examination revealed diaphoretic ingress, tachycardic, afebrile, semidry mucous membranes, disoriented, disintegrated thought, hypoprosexic, bradylaliac, glabellar reflex present, with generalized rigidity, and mild tremor in the upper limbs.

Paraclinical exams at admittance are reported in Table 1, EKG is taken with only abnormality QTc 500 msec, simple skull CT scan within normal limits; given the alterations of consciousness associated with increased CPK and stiffness, a NMS diagnosis was made; patient is taken to the resuscitation area, initiating management with Ringers Lactate 2 cc/kg with target urine output of 2 cc/kg/hour, Lorazepam 2 mg orally every 8 hrs, and Bromocriptine 2.5 mg every 12 hours, monitoring thermal curve hourly, daily renal function monitoring, daily CPK control, and urine output.

Patient presents adequate evolution of symptoms of admittance, with complete disappearance of muscle stiffness, improved state of consciousness, no episodes of hyperthermia during hospitalization, with adequate urine output, and decreased CPK without compromise of renal function (Table 2) so that counter-referral to a psychiatric clinic where it was being handled was made.

## 3. Discussion

The Neuroleptic Malignant Syndrome (NMS) was first described in 1960 [1, 2]; it has a low incidence (0.5 to 3%) in patients with use of neuroleptic drugs [3], having an increased risk with typical antipsychotic drugs versus atypical antipsychotics, with mortality reaching 10% [4, 5]. The pathophysiological mechanism is not precise. It is believed to be the result of altered dopamine transmission and, in some cases, it has a direct effect on skeletal muscle [6].

There is a blockage of the dopaminergic pathways, explaining the development of signs and symptoms of this syndrome as parkinsonism, rigidity, tremor, autonomic dysfunction, hyperthermia, and instability of cardiorespiratory parameters [7]. The two pathogenic mechanisms are as follows: the first mechanism is reduction of dopaminergic signals as a result of acute withdrawal or withdrawal of dopaminergic agents [6]; the other mechanism is by blocking the dopamine signal with the use of medications such as metoclopramide and neuroleptics [8]. The increase in epinephrine and serotonin contributes to the alteration in the transmission of dopamine [9], contributing to hyperthermia and rigidity [6]. There is rhabdomyolysis and muscle damage especially with the use of chlorpromazine and fluphenazine [10].

The defining characteristics of NMS are hyperthermia, motor symptoms, altered mental status, and autonomic instability; the first two constitute the clinical marker of the syndrome.

Early symptoms are tachycardia, arrhythmias, hypotension, and cardiac arrest. 50–80% of patients have muscle stiffness, mainly in the form of lockjaw. Hyperthermia usually occurs later and in final phases pulmonary edema and cerebral and disseminated intravascular coagulation occur [11, 12].

The diagnosis is made by combining clinical and laboratory findings, using the criteria of DSM-V [13] and Levenson [14].

Despite different meta-analysis, there is no consensus that has standardized treatment; suspending the dopaminergic blocking agent or initiating the suspended agonist, hydration, decrease in temperature, correction of electrolyte abnormalities, thromboprophylaxis, and drug treatment as dopamine agonists (bromocriptine as first choice) are suggested, as well as muscle relaxants such as dantrolene. In some cases, ventilatory support or dialysis is suggested until the patient regains cardiorespiratory and renal functions and presents CPK concentrations of <1,000 U/L functions.

Associated acute renal failure is considered the most serious complication [15] and it is an independent predictor of mortality [16]; this is due to the intense rhabdomyolysis, deposit of myoglobin in the renal tubules, and dehydration, which occurs in about 16% of cases [17] with a mortality of 50% [18].

## 4. Conclusion

NMS is an uncommon and lethal neurological disorder [19], attributed to the administration of typical antipsychotics [2, 6, 8, 20, 21] and less commonly to the atypical antipsychotics, like clozapine [5], which has a direct dopaminergic effect but in less proportion than typical antipsychotics especially in D2 receptors. However, clozapine has an effect over serotoninergic pathways that stimulate the 5-HT_1A_ receptor (5-hydroxytryptamine) causing recirculation of gamma amino butyric acid in the nucleus accumbens leading to the inhibition of the dopaminergic neurons [22], related to the development of the disease. There are also some described cases in which the medication relates with stiffness and fever but less frequently [12, 23].

In our particular case, this diagnosis was considered, since it had rigidity, altered state of consciousness, dysautonomia, and elevation of CPK, with a predisposing pharmacological condition, based on the clinical criteria of DSM-V and Levenson, without different drug consumption in the last 10 years of treatment, evolving appropriately with benzodiazepine, hydration, and withdrawal of clozapine, after 5 days of treatment initiation. The patient may have benefited from the use of dantrolene which was not administered because of the nonavailability of this drug in the institution.

However, we emphasize the absence of hyperthermia, since its maximum temperature registered was 37.3°C, fever being one of the typical criteria found in this syndrome [24] considering it as an atypical variant [25].

## Figures and Tables

**Table 1:** 

	Result	Control
Leucocytes (10^3^)	7,2	5.0–10
Neutrophils (%)	63,9	45–70
Lymphocytes (%)	23	20–45
Hemoglobin (gr/dl)	15,9	11–16,5
Hematocrit (%)	46	42–52
Platelets (10^3^)	224.9	150–450
VSG (mm/hour)	21	0–20
PCR (mg/dl)	1,2	<2
Glycemia	95	65–110
Sodium (mEq/l)	140	137–145
Potassium (mEq/l)	3,4	3,6–5
Chlorine (mEq/l)	110	98–107
Creatine kinase (CPK) (U/l)	12446	0–50
Creatinine (mg/dl)	0,88	0,52–1,3
Ureic nitrogen (mg/dl)	24,5	7,0–20

**Table 2:** 

	Day 1	Day 2	Day 3	Day 4	Day 5	Day 6
Creatine kinase (CPK)	12,446 U/l	11,778 U/l	9,046 U/l	8,300 U/l	2,762 U/l	2,245 U/l
Creatinine (mg/dl)	0,84	0,81	0,64	0,71	0,64	0,63
Temperatures	36,4–37,1°C	36,5–37°C	36,7–37,1°C	36,5–37°C	36,4–37,3°C	36,6–37°C
Urine output	1,3 cc/kg/hour	2,4 cc/kg/hour	2,8 cc/kg/hour	2,0 cc/kg/hour	1,8 cc/kg/hour	2,0 cc/kg/hour
